# A Rare Cause of Primary Amenorrhea in Adolescence: A Case Report

**DOI:** 10.7759/cureus.89760

**Published:** 2025-08-10

**Authors:** Alexandra Rodrigues, Lara Torres, Rita Salgueiro, Isabel González, Rita S Oliveira

**Affiliations:** 1 Department of Pediatrics, Unidade Local de Saude da Guarda, Guarda, PRT

**Keywords:** absent uterus, adolescent medicine, holistic evaluation, mayer-rokitansky-küster-hauser (mrkh) syndrome, primary & secondary amenorrhea

## Abstract

Primary amenorrhea is the absence of menses at age 15 with secondary sexual characteristics or at age 13 without any secondary sexual characteristics. Evaluation for primary amenorrhea should also be considered when menarche has not occurred within three years of thelarche, or five years after thelarche if it began before the age of 10. We report a rare case of a 15-year-old adolescent with primary amenorrhea who was diagnosed with Mayer-Rokitansky-Küster-Hauser (MRKH) syndrome. The diagnosis was established through magnetic resonance imaging. The patient is currently undergoing multidisciplinary follow-up, including psychological support. At present, the use of vaginal dilators or surgical intervention is being considered as the most appropriate therapeutic approach.

## Introduction

Primary amenorrhea is defined as the absence of menstruation by age 15 in the presence of secondary sexual characteristics, or by age 13 in their absence [1]. It can result from dysfunction in one or more components of the hypothalamic-pituitary-ovarian-uterine axis, including the hypothalamus, pituitary gland, ovaries, uterus, or vagina [1,2,3]. Evaluation for primary amenorrhea should also be considered if menarche has not occurred three years after thelarche, or five years after thelarche if it began before the age of 10 [1]. The most common etiology of primary amenorrhea is gonadal dysgenesis, in which Turner syndrome is included [3].

In the evaluation of an adolescent with primary amenorrhea, a thorough medical history and detailed physical examination are essential to identify potential symptoms or past events that may raise clinical suspicion for a specific diagnosis. For instance, a history of adrenal crisis in the neonatal period suggests the possibility of congenital adrenal hyperplasia. A lifestyle involving intense physical activity or disordered eating behaviors may point toward hypothalamic amenorrhea [1,2,3]. On physical examination, signs of hyperandrogenism-such as acne, hirsutism, or a Ferriman-Gallwey score greater than eight-should prompt consideration of differential diagnoses, including polycystic ovary syndrome, five-alpha-reductase deficiency, or androgen-producing tumors [3]. Conversely, findings such as short stature, a broad neck, and increased internipple distance may indicate Turner syndrome. Following a comprehensive history and physical examination, complementary diagnostic tests should be guided by the leading clinical hypothesis. Nevertheless, it is essential to obtain laboratory investigations, including a complete hormonal profile, and perform imaging studies-initially pelvic ultrasonography only to confirm the suspected diagnosis when present, but also to exclude other, more prevalent etiologies [1,2,3].

It is also important to recognize that rarer conditions may underlie primary amenorrhea. Maintaining clinical suspicion and ensuring an early, multidisciplinary approach can enable timely diagnosis and management. Examples include Mayer-Rokitansky-Küster-Hauser (MRKH) syndrome, Kallmann syndrome, or 17 hydroxylase deficiency, among others [1,2].

This article was previously presented as a meeting abstract at the European Academy of Pediatric Societies (EAPS), held on October 7-11, 2022.

## Case presentation

A 15-year-old adolescent undergoing follow-up for asthma and rhinitis in pediatric allergology was found to have primary amenorrhea. During the evaluation, it was noted that her mother had experienced menarche at age 15, and no other relevant findings were identified in the patient’s medical history. On physical examination, she appeared overweight, with a weight between the 50th and 85th percentiles and a height at the 15th percentile. No other particularities were noted. She was at Tanner stage IV, had a non-palpable thyroid gland, and a Ferriman-Gallwey score of 6 (<8). An extensive laboratory workup was requested, including follicle-stimulating hormone (FSH), luteinizing hormone (LH), thyroid-stimulating hormone (TSH), free thyroxine (T4), cortisol, progesterone, estradiol, and dehydroepiandrosterone sulfate (DHEA-S), along with an abdominal ultrasound. The laboratory results were entirely within normal limits (Table 1). The ultrasound described: “slightly globular ovaries with some cystic structures; a few interrupted segments of a rudimentary uterine area, to be further evaluated by pelvic magnetic resonance imaging (MRI) for accurate locoregional characterization.” The pelvic ultrasound raised uncertainty regarding the presence or absence of the uterus. Given that the laboratory analysis excluded hormonal abnormalities, the differential diagnoses were anatomical, including outflow tract obstruction such as imperforate hymen, transverse vaginal septum, distal vaginal atresia, or cervical agenesis. In cases of absent uterus, Müllerian agenesis or androgen insensitivity syndrome were also considered. Therefore, a pelvic MRI was requested for further clarification.

**Table 1:** Analytical study performed.

Analysis		Reference values
Follicle-stimulating hormone (FSH)	1.85 mUI/mL	Follicular: 3.03-8.08; ovulation: 2.55-16.69; luteal: 1.38-5.47
Luteinizing hormone (LH)	2.98 mUI/mL	Follicular: 1.80-1.78; ovulation: 7.59-89.08; luteal: 0.56-14.00
Progesterone	1.40 ng/mL	Follicular: 0.2-0.3; ;uteal: 1.2-15.9
Estradiol	82 pg/mL	Follicular: 21-251; ovulation: 38-649; luteal: 21-312
Dehydroepiandrosterone-sulfate (DHEA-S)	162 µg/dL	8.60-169.80
Thyroid-stimulating hormone (TSH)	0.412 µUI/mL	0.350-4.940
Thyroxine (T4 free)	0.9 ng/dL	0.7-1.5

**Figure 1:**
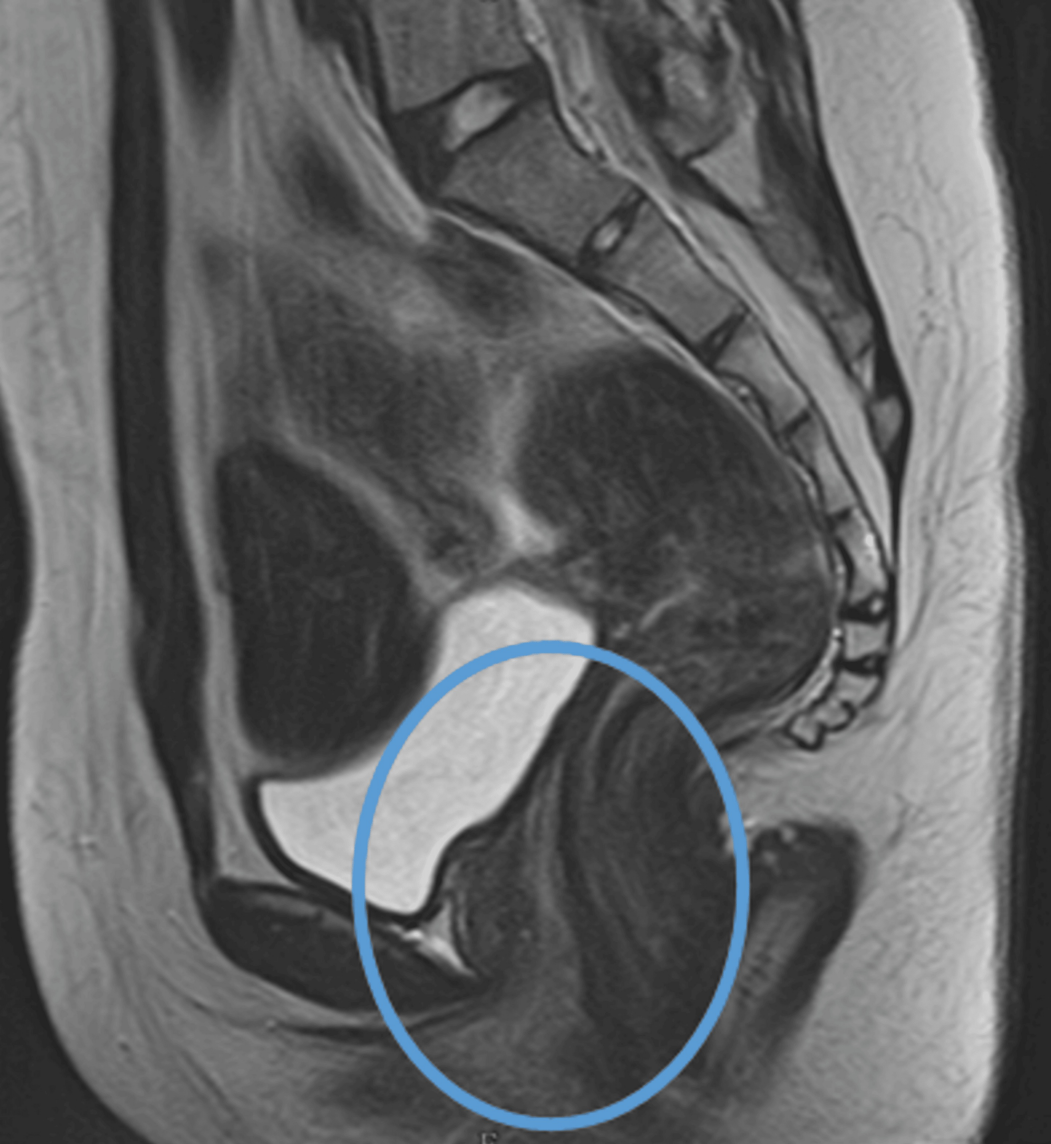
Pelvic magnetic resonance imaging: sagittal section. The circled area highlights the location where the uterus is absent and the vagina ends in a blind pouch.

**Figure 2:**
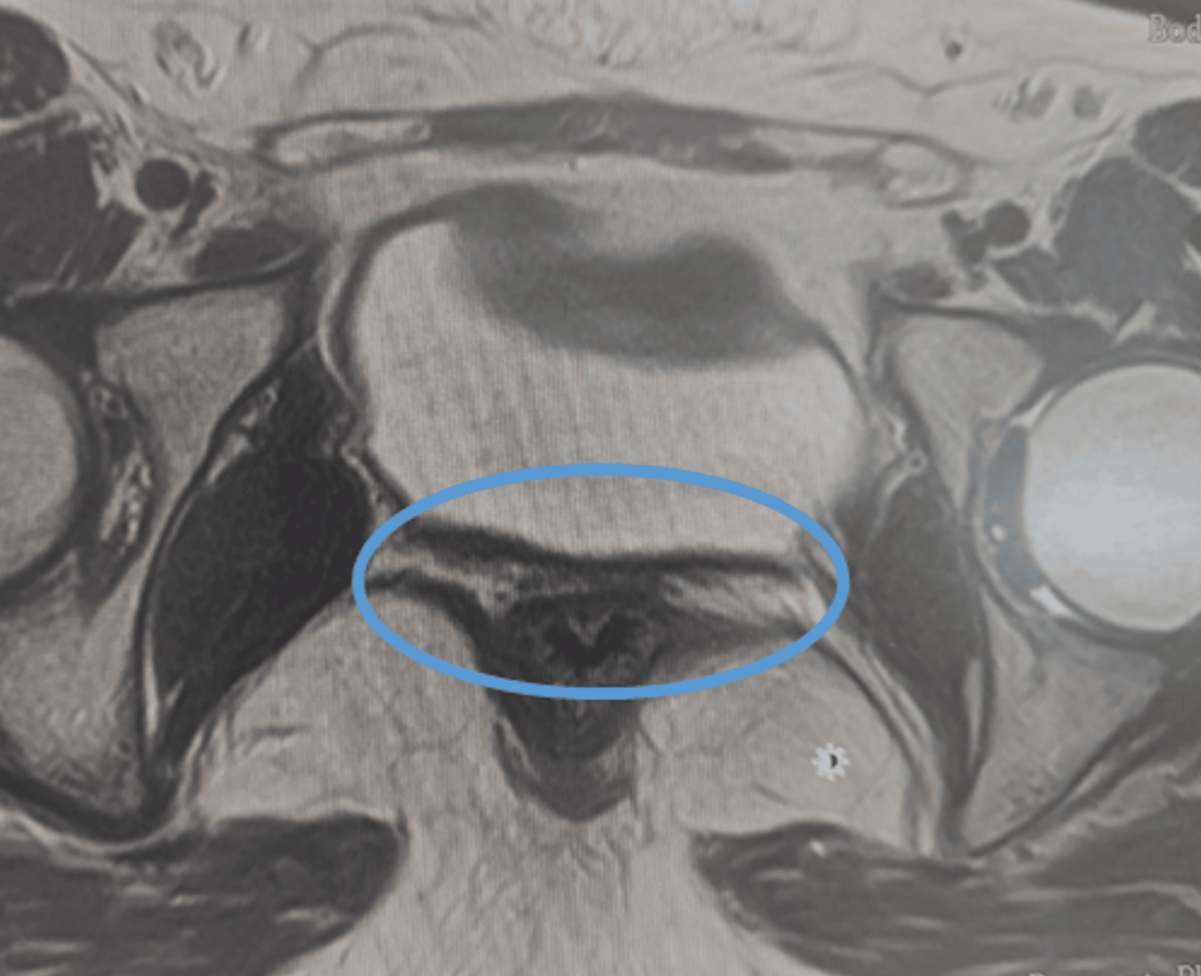
Pelvic magnetic resonance imaging: axial section. The circled area highlights the location where the uterus is absent.

The MRI report stated: “Unidentified uterus. Ovaries with normal characteristics” (Figures 1-2). Based on these findings, primary amenorrhea was attributed to uterine agenesis, and a diagnosis of MRKH syndrome was established. Her karyotype was 46, XX, and an endocrinology and gynecology referral was made. The patient also underwent abdominal and renal ultrasonography to exclude nephrourological malformations. The results were unremarkable, allowing the diagnosis to be classified as type 1 MRKH syndrome.

The patient is currently receiving multidisciplinary follow-up care involving an allergologist, pediatrician, endocrinologist, gynecologist, and psychologist. A genetic evaluation was also requested to assess for any identifiable mutations. At the endocrinology consultation, an extended hormonal panel was requested, including androstenedione, 17-hydroxyprogesterone, cortisol, and adrenocorticotropic hormone (ACTH); all results were within normal limits. The patient is now seen annually by endocrinology. During the gynecological consultation, the external genitalia appeared normal, with a well-formed vulva and intact hymen. Upon gentle insertion of a cotton swab, a narrow vaginal canal measuring approximately 2-2.5 cm in length was identified. As such, the use of vaginal dilators or surgical intervention was proposed as a possible therapeutic option. The final decision will be made according to the adolescent’s preferences, following comprehensive counseling on the benefits, risks, and implications of each option.

## Discussion

Primary amenorrhea affects approximately 0.1% of all adolescents [4]. Its underlying causes include endocrine disorders, chronic illnesses, genetic mutations, and Müllerian duct anomalies [3,4]. Etiologies can be broadly classified into anatomical and sexual development abnormalities, ovarian insufficiency, hypothalamic or pituitary disorders, and other endocrine gland dysfunctions. Although certain medications can cause amenorrhea, they are more frequently implicated in secondary amenorrhea [5].

MRKH syndrome has a reported prevalence of 1 in 5,000 females, with estimates ranging from 1 in 4,000 to 1 in 10,000 [3,6,7]. This syndrome is characterized by congenital absence or underdevelopment of the uterus and variable development of the vagina. Urologic anomalies are present in 25%-50% of cases and may include unilateral renal agenesis, pelvic or horseshoe kidneys, or abnormalities of the collecting system [3,8]. MRKH syndrome is classified as type 1 when there is isolated uterovaginal aplasia (affecting organs derived from the Müllerian ducts), and as type 2 when associated anomalies are present, including renal or skeletal malformations [9].

The etiology of MRKH remains unclear, but several genetic mutations have been identified, including WNT3 (Wnt Family Member 3), HNF1B (hepatocyte nuclear factor 1-beta), and LHX1 (LIM homeobox 1) [3,6,7,8]. Since ovarian development and function are typically normal, affected adolescents usually present between the ages of 15 and 17 with primary amenorrhea, as secondary sexual characteristics develop normally [7,10]. These patients typically have normal laboratory results, and diagnosis relies primarily on imaging, including ultrasound and MRI [3,7,8].

The first-line treatment is non-invasive, using vaginal dilators. However, surgical interventions such as vaginoplasty for neovagina creation or even uterine transplantation may be considered, particularly in cases where dilator therapy fails or when urological anomalies are present [3,7,8,10].

Adolescents with MRKH often require multidisciplinary management, including psychological support, given the emotional and psychosocial burden associated with the diagnosis. The condition entails the absence of menstruation, potential difficulties with sexual activity, and infertility, all of which can be distressing for young patients and negatively impact their emotional well-being [8].

This article presents a rare case of primary amenorrhea with a final diagnosis of MRKH syndrome, established through pelvic MRI. MRI was essential not only for confirming the diagnosis but also for excluding associated malformations, which is consistent with the literature. The patient was diagnosed with type 1 MRKH, characterized by isolated uterovaginal aplasia. The main differential diagnoses for type 1 MRKH syndrome include complete androgen insensitivity syndrome, isolated vaginal agenesis or atresia, cervical agenesis, uterine hypoplasia, and outflow tract obstructions such as imperforate hymen or transverse vaginal septum [1,2,3,7,8]. In this case, these conditions were effectively excluded based on thorough physical examination, normal analytical study, and an MRI without a uterus identified. The therapeutic approach is currently under consideration-starting with vaginal dilators and reserving surgical intervention for refractory cases or more complex malformations, as recommended by the literature. This adolescent is presently receiving multidisciplinary care to optimize her quality of life.

## Conclusions

In conclusion, primary amenorrhea encompasses a wide spectrum of possible diagnoses, ranging from easily treatable to more complex conditions. A thorough medical history, comprehensive physical examination, appropriate laboratory investigations, and imaging are essential for timely and accurate diagnosis. This case highlights the importance of a holistic clinical approach, even when the initial consultation is for unrelated concerns - as in this instance, where the first evaluation was initiated by a pediatric allergologist.
